# Electrical Oscillations in Two-Dimensional Microtubular Structures

**DOI:** 10.1038/srep27143

**Published:** 2016-06-03

**Authors:** María del Rocío Cantero, Paula L. Perez, Mariano Smoler, Cecilia Villa Etchegoyen, Horacio F. Cantiello

**Affiliations:** 1 Cátedra de Biofísica, Facultad de Odontología. Universidad de Buenos Aires, Buenos Aires, Argentina

## Abstract

Microtubules (MTs) are unique components of the cytoskeleton formed by hollow cylindrical structures of αβ tubulin dimeric units. The structural wall of the MT is interspersed by nanopores formed by the lateral arrangement of its subunits. MTs are also highly charged polar polyelectrolytes, capable of amplifying electrical signals. The actual nature of these electrodynamic capabilities remains largely unknown. Herein we applied the patch clamp technique to two-dimensional MT sheets, to characterize their electrical properties. Voltage-clamped MT sheets generated cation-selective oscillatory electrical currents whose magnitude depended on both the holding potential, and ionic strength and composition. The oscillations progressed through various modes including single and double periodic regimes and more complex behaviours, being prominent a fundamental frequency at 29 Hz. In physiological K^+^ (140 mM), oscillations represented in average a 640% change in conductance that was also affected by the prevalent anion. Current injection induced voltage oscillations, thus showing excitability akin with action potentials. The electrical oscillations were entirely blocked by taxol, with pseudo Michaelis-Menten kinetics and a *K*_*D*_ of ~1.29 μM. The findings suggest a functional role of the nanopores in the MT wall on the genesis of electrical oscillations that offer new insights into the nonlinear behaviour of the cytoskeleton.

MTs play important roles in cell functions, including acting as railways for motor proteins, vesicles and organelles, and separating chromosomes during cell division^1^,^2^,^3^. MTs are unique among the components of the cytoskeleton, because they are hollow tubes made from αβ tubulin heterodimeric subunits stacked head to tail into protofilaments^4^,^5^,^6^. Most often 13 protofilaments are incorporated in an MT wall where neighbouring subunits have a slight three-subunit longitudinal shift along each other, although other arrangements with macrotubules of larger diameters are also observed^6^. MTs form intracellular superstructures of variable complexity and defined biological role(s) in cell function. The mitotic spindle, for example, is a large dynamic array of MTs that segregates chromosomes and orients the plane of cleavage during cell division. The axonemal structure of sensory and motile cilia and flagella contain nine doublets of MTs, and triplets of MTs are observed in centrioles and basal bodies. A relevant intermediary between the αβ tubulin subunit and the structurally cylindrical MT is a two-dimensional sheet of protofilaments that bind laterally one another prior to eventually bending into a helical form^7^,^8^,^9^,^10^,^11^,^12^. Two-dimensional MT sheets make an important contribution to the dynamic structure of MT assembly *in vivo*^11^,^12^,^13^. Stable sheets of protofilaments show distinct structural lattices dependent on the lateral interactions between adjacent subunits^6^. These lateral arrangements generate at least two types of nanopores along their connecting interface, where one nanopore is formed between adjacent αβ interdimer interfaces, and another smaller type, apparently arises between αβ intradimer interfaces^6^,^14^. Because MTs produce mechanical oscillations^15^,^16^,^17^,^18^, involving both transversal, as well as radial motions^19^,^20^, it is entirely possible that mechanical changes affect the topological features of the nanopores and thus their permeability properties, if any. MTs are also highly charged electrically polarized polymers, where αβ tubulin heterodimers have a high electric dipole moment^21^ that is manifested by the MT’s high sensitivity to electric fields both *in vitro*^22^,^23^ and *in vivo*^24^. MTs present extremely large uncompensated charge^25^, and electrostatic interactions may play an important role in MT macromolecular assembling and function^26^. Thus, MTs generate oscillatory electric fields at expense of elasto-electrical vibrations in the tubulins^27^. These electrical properties may be at the centre of the ability of the MT to amplify and axially transfer electrical signals^28^,^29^, and thus behave as a sophisticated nonlinear transmission line^30^,^31^,^32^,^33^. To date, however, no experimental evidence has been gathered as to the molecular aspects of the electrical signals generated by MTs.

Herein we attempted to bridge this gap by exploring the electrical properties of flat two-dimensional MT sheets, a macroscopic equivalent of the MT wall^5^,^34^,^35^, with the conventional patch clamping technique^36^. We improved on the method for patching MTs^28^,^29^, obtaining conditions to reach gigaseal resistances in MT sheets that disclosed detailed electrical properties of the preparations tested. We observed rather remarkable electrical oscillatory behaviour of the MT sheets, which we describe qualitatively in this first communication. The voltage-clamped MT sheets displayed current oscillations that changed in amplitude and oscillatory response, depending on both holding potential, and ionic strength and composition. Conversely, current clamped MT sheets displayed voltage oscillations that resembled the transient changes in conductance observed in action potentials. In considering the various structures based on MTs, the novel electrodynamic properties of the MTs presented herein may offer a new dimension in the complexity of cytoskeletal structures.

## Results

### MT sheets from different origins

**Figure 1:**
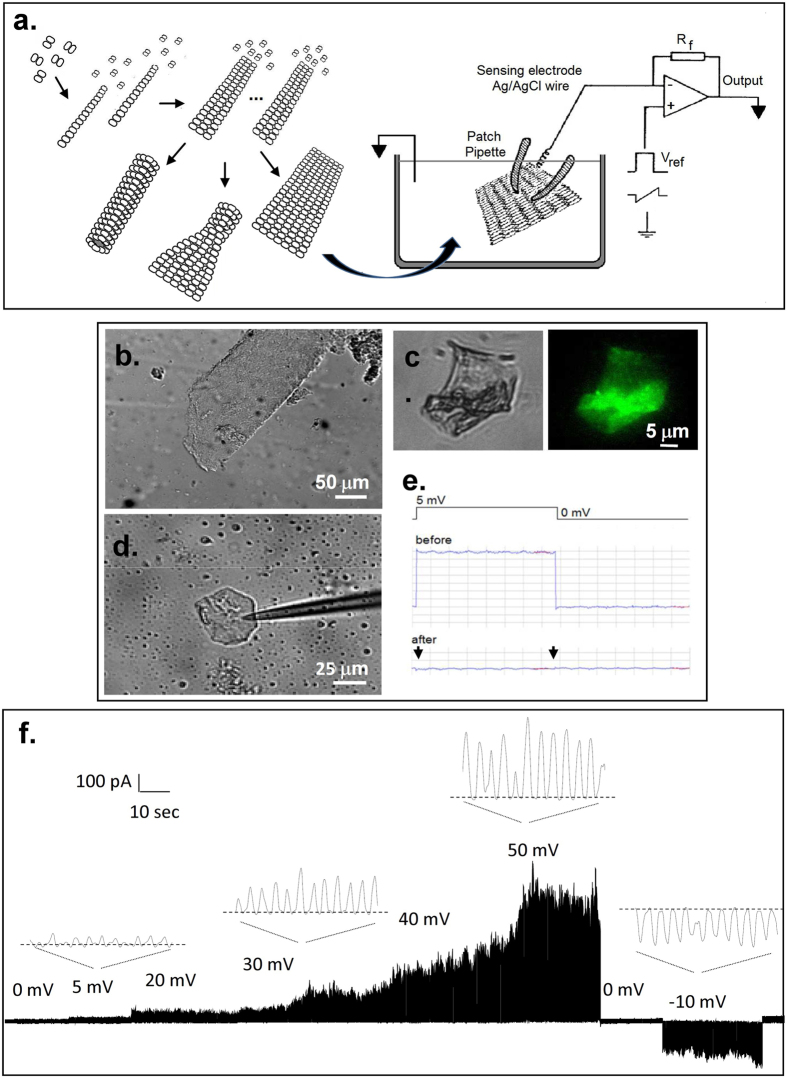
Microtubular sheets and experimental approach. (**a)** *Left*. Different tubulin conformations observed in preparations towards the formation of MTs. *Right*. Schematics of the conventional patch clamp setup to obtain electrical properties of MT sheets. (**b)** MT sheet obtained from a bovine brain microtubular preparation (DIC x20). Similar sheets were also obtained from rat brain and commercial tubulin. (**c)** Immunochemical labelling of MT sheet with anti-tubulin antibody. (**d)** A patch pipette was used to connect the MT sheet to a patch clamp amplifier. Shown is an APTES-attached MT sheet connected to a patch pipette (DIC x20). (**e)** Electrical recording of patch formation was followed with a 5 mV 500 ms long square pulse. The patch pipette in 140 mM KCl solution had a tip resistance of 14 MΩ that increased to 1.4 GΩ after touching the MT sheet. It is noticeable the lack of capacitive current after sealing (arrows). (**f)** Time series recording of a free-floating MT sheet from bovine brain with a 780 MΩ seal resistance without APTES, to which several holding potentials, both positive and negative are imposed. Expanded tracings show the oscillatory phenomenon observed that changed in amplitude with the magnitude and polarity of the holding potential, but absent at zero mV in symmetrical KCl. Oscillations change direction with voltage polarity.

The experiments described herein were conducted with MT sheets obtained either from cow or rat brain, or made directly from purified (bovine brain) commercial tubulin, rendering rather similar qualitative results. All three preparations spontaneously formed large MT sheets (Fig. 1b). MT sheets from both rat and cow brains were readily observed in partially isolated MT preparations (one polymerization-depolymerization cycle), suggesting their presence during the isolation procedure. The presence of tubulin in the MT sheets was confirmed by immunochemistry with anti-α tubulin antibody (Fig. 1c).

### Electrical properties of MT sheets

To improve on the electrical information previously reported with isolated, taxol-stabilized MTs^28^,^29^, MT sheets were instead patched in this study under taxol-free conditions (see Methods and Fig. 1a) with the conventional patch clamping technique^36^. The surface was approached with a patch pipette connected to a patch clamp amplifier (Fig. 1a,d,e). Experiments were usually initiated under symmetrical conditions, with an “intracellular” high K^+^, Ca^2+^-free solution containing 1 mM EGTA (see Methods). Apposition of the pipette tip onto the MT sheet usually increased the resistance to 30–50 MΩ (Table 1). High resistance (>100 MΩ) seals on free-floating MT sheets made of purified tubulin were seldom observed (Table 1). An example of a 780 MΩ sealed patch showing the main features of the findings is observed in Fig. 1f. A major improvement in data acquisition was achieved by seeding MT sheets onto APTES-coated glass surfaces. MT sheets thus stabilized were easier to patch and rendered much higher resistance seals in almost every attempt made (Table 1). Sealed MT sheets displayed spontaneous electrical activity consistent with “multi channel-like” behaviour (Fig. 1f), in that currents responded to holding potentials with transient changes in conductance, however, inconsistent with “quantum” jumps observed in classical ion channel fashion, namely, extremely fast on-off rates from stochastic transitions between open and closed states, but rather showing highly synchronized, self-sustained electrical oscillations that responded directly to the magnitude of the stimulus. All three MT sheet preparations displayed almost identical features, suggesting that neither contaminant proteins present in the less purified preparations nor the various technical procedures for MT isolation and MT sheet formation had any noticeable effect on the electrical behaviour observed. Addition of Zn^2+^ ions induces large assemblies of tubulin protofilaments^8^ arranged with alternating antiparallel polarity rather than parallel arrays of protofilaments that constitute normal MTs. The strong similarity in the oscillatory response between preparations, however, suggests that it is not a Zn^2+^-induced conformational state that is studied. This is consistent with the fact that the images of the subunits in Zn^2+^-induced sheets agree well with those obtained from normal sheets, or opened-out MTs, supporting that inter-protofilament spacing is essentially identical in both types of sheet^37^.

### Time and frequency domain analyses

**Figure 2:**
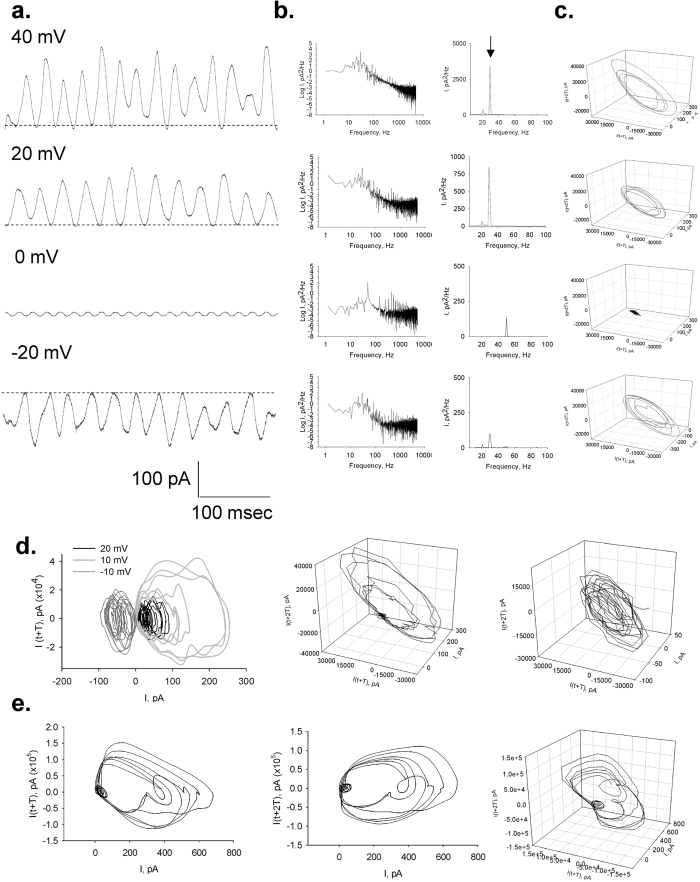
Current oscillations of voltage-clamped MT sheets. (**a)** Representative tracings of the effect of the holding potential on MT sheet current oscillations. Data were obtained by applying 1.5-sec voltage steps in symmetrical 140 mM KCl. (**b)** Log-Log, and Linear-linear detail of Fourier power spectra obtained from unfiltered current responses of tracings on (**a**), showing the fundamental 29 Hz oscillatory frequency. No peak is observed at zero mV but instead appears a prominent 50 Hz signal due to line contamination. (**c)** Three-dimensional phase-space portraits showing monoperiodic limit cycles. There is a tendency to period doubling at higher holding potentials. Delay time for first and second derivatives adopted for phase portraits was 1 ms. (**d)** *Left*, limit cycles for successive 10 mV (Light Gray), −10 mV (Dark Gray) and 20 mV (Black) holding potentials, showing a later aspect of the time series even more chaotic-type of behaviour at that negative potential. This is more evident in the phase portraits for positive (*Centre*) and negative (*Right*) potentials. (**e**) *Left* and *Centre*, the more complex behaviour at negative potential slowly returns to more stable cycles upon return to positive holding potential of similar magnitude (*Right*).

**Figure 3:**
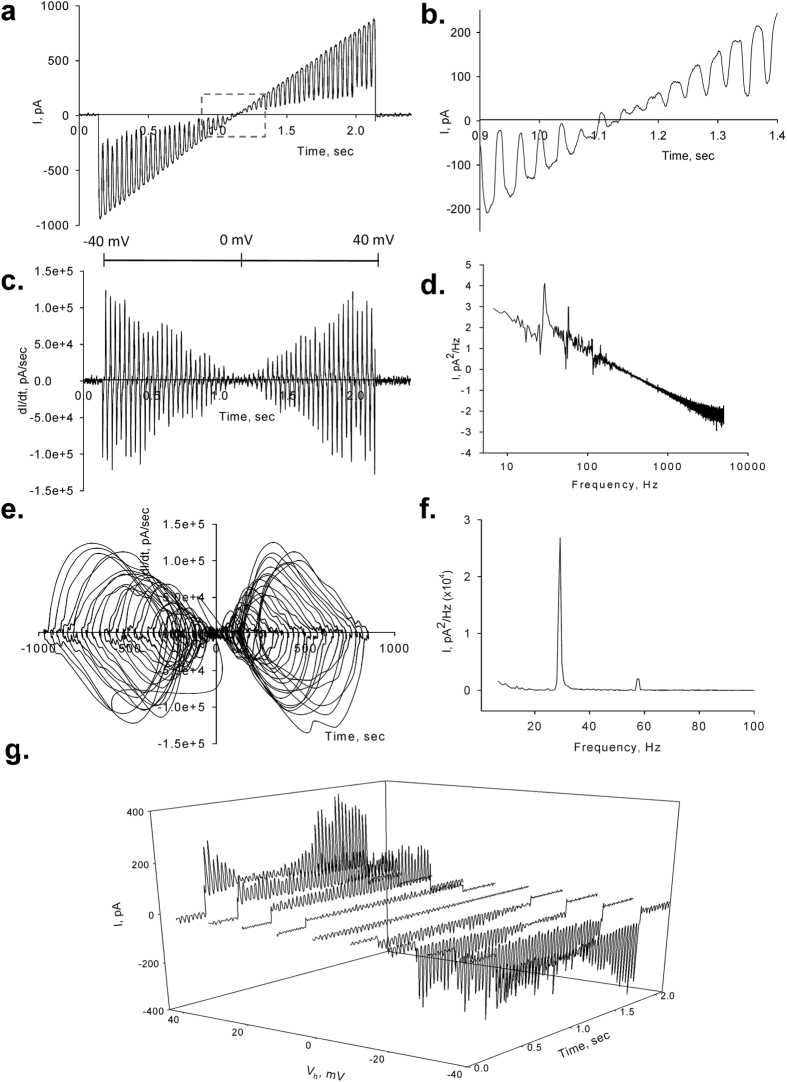
Effect of rapid voltage changes on the current oscillations. Current oscillations were also obtained at instantaneous holding potentials applied from ramps of 2 ms. (**a)** Current oscillations between ±40 mV showed linear amplitude. (**b)** Expanded detail from square box in (**a)** shows the reversal potential at zero mV and the immediate appearance of oscillations both at negative and positive potentials. (**c)** Time delay derivative of *dI/dV* shows the increase in amplitude *dI/dt* for both negative and positive potentials, indicating cycling stability at 29 Hz, as shown in the Fourier transformation in (**d)**. (**e)** Limit cycles of *dI/dt* showing the monoperiodic behaviour of the oscillations. (**f)** Linear-linear detail of the Fourier transformation showing the 29 Hz fundamental frequency. (**g)** Three-dimensional response of a train of successive 1.5 sec voltage steps between ±40 mV showing changes in amplitude not observed with ramp protocols. Lag time between voltage steps is 0.5 sec.

**Figure 4:**
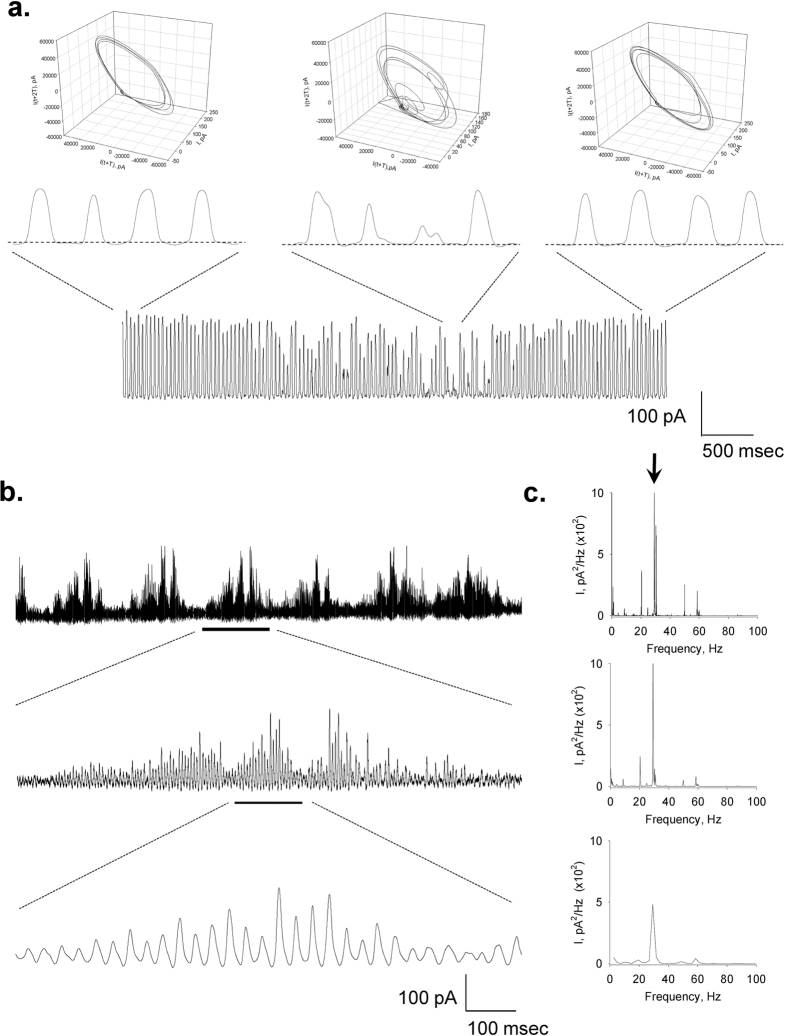
Changes in oscillatory behaviour. The oscillatory behaviour of a patched MT sheet showed various spontaneous regimes without any changes in the driving forces. (**a)** Short periods of chaotic behaviour could be observed in pseudo-monoperiodic regimes as shown in the expanded sections. These changes are also observed in the phase portraits shown above each tracing. (**b)** Periodic changes in the amplitude of the oscillations showed fractal “envelopes” of increasingly lower frequency. (**c)** Power spectra for respective tracings in (**b**) show the fundamental frequency at 29 Hz.

**Figure 5:**
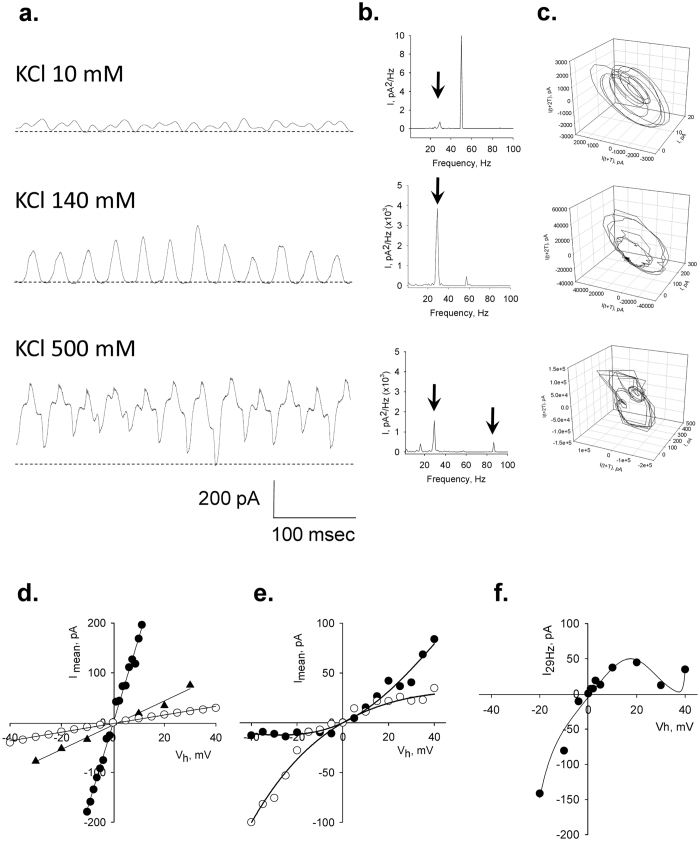
Effect of ionic strength on the electrical oscillations of MT sheets. (**a)** Oscillatory currents were observed at different KCl symmetrical concentrations, including 10 mM, 140 mM, and 500 mM. The simplest monoperiodic response was observed closer to physiological concentration of KCl. Both amplitude and frequency responses were different although the 29 Hz fundamental frequency was observed at the various concentrations as shown in the Linear-linear Fourier transform (**b**). All concentrations presented cycle doubling and more complex behaviour at 500 mM KCl. (**c)** Three-dimensional phase-space portraits showing monoperiodic limit cycles. There is a tendency to period doubling at higher holding potentials. Delay time (*T*) for first and second derivatives adopted for phase portraits was 1 ms. (**d)** The current-to-voltage relationships obtained from integrated, mean, currents of individual experiments, were highly linear (open circles, triangles, and filled circles representing lower to higher concentration, respectively) although longer time series elicited strong rectification both inward and outward (**e**) without any changes in electrochemical gradient. Data were successfully fitted with a 2S3B energy model (Black lines). (**f)** The current-to-voltage relationship of the 29 Hz peak also showed strong rectification. Experimental data were fitted with a tunnel diode current equation (see text).

To observe the properties of the electrical oscillations, patched MT sheets were initially voltage-clamped at a holding potential of zero mV in symmetrical KCl solution (140 mM) showing no apparent electrical activity. Any departure from electrochemical equilibrium readily manifested oscillatory behaviour. The initial oscillatory response most frequently consisted of a monoperiodic regime often showing regular sequences of smaller and larger peaks (Figs 1f and 2a), and a remarkably stable fundamental frequency of 29 Hz observed at different holding potentials (Fig. 2b). More complex behaviours were often observed without any deliberate change in driving force (Fig. 2c), and several oscillatory states emerged during lengthy runs at constant electrochemical conditions. Three-dimensional phase portraits constructed with the time delay method showed the period doubling in the single limit cycles, and more complex behaviours at negative potentials (Fig. 2c–e). Electrical currents were also obtained with ±40 mV voltage ramp protocols (Fig. 3a–f). Derivation of the conductance allowed the *dI*/*dt* response from which the Fourier transform confirmed the monoperiodic cycle at 29 Hz (Fig. 3d,f) and voltage independence of the fundamental frequency. Instead, two-second voltage step protocols in the same range manifested more complex behaviours with changes in both amplitude and oscillatory cycling (Fig. 3g). Under these conditions, a maximal change in the variable conductance of 643% was obtained, by calculating the difference between basal (non-oscillatory), and peak current conductances (1.82 ± 0.40 vs. 11.47 ± 2.08 nS, n = 7, respectively, p = 0.0015). Noticeable oscillatory changes such as sudden death disappearance of oscillations (Fig. 4a) and multiple amplitude (fractal type) oscillatory responses (Fig. 4b) were prominent in some experiments. A linear mean conductance was observed from voltage step protocols at all concentrations tested (Fig. 5d), including 10 mM (0.86 nS), 140 mM (2.4 nS) and 500 mM symmetrical KCl (17 nS) (Fig. 5a,d), although higher KCl concentration (500 mM) also showed more complex oscillatory behaviour (Fig. 5c, Bottom) and the presence of other fundamental frequencies (Fig. 5b, Bottom). However, under symmetrical KCl conditions (Fig. 5e) the mean conductance often showed both inwardly and outwardly rectifying properties in the same patch. Interestingly, the peak conductance obtained at the 29 Hz fundamental frequency, also showed strong nonlinearity (Fig. 5f), despite a mean linear response. This nonlinear electrical response showed a clear negative resistance region that was modelled with an Esaki diode current equation (Equation 2) displaying a “tunnelling” effect (see Methods).

### Ionic selectivity of the transport process

**Figure 6:**
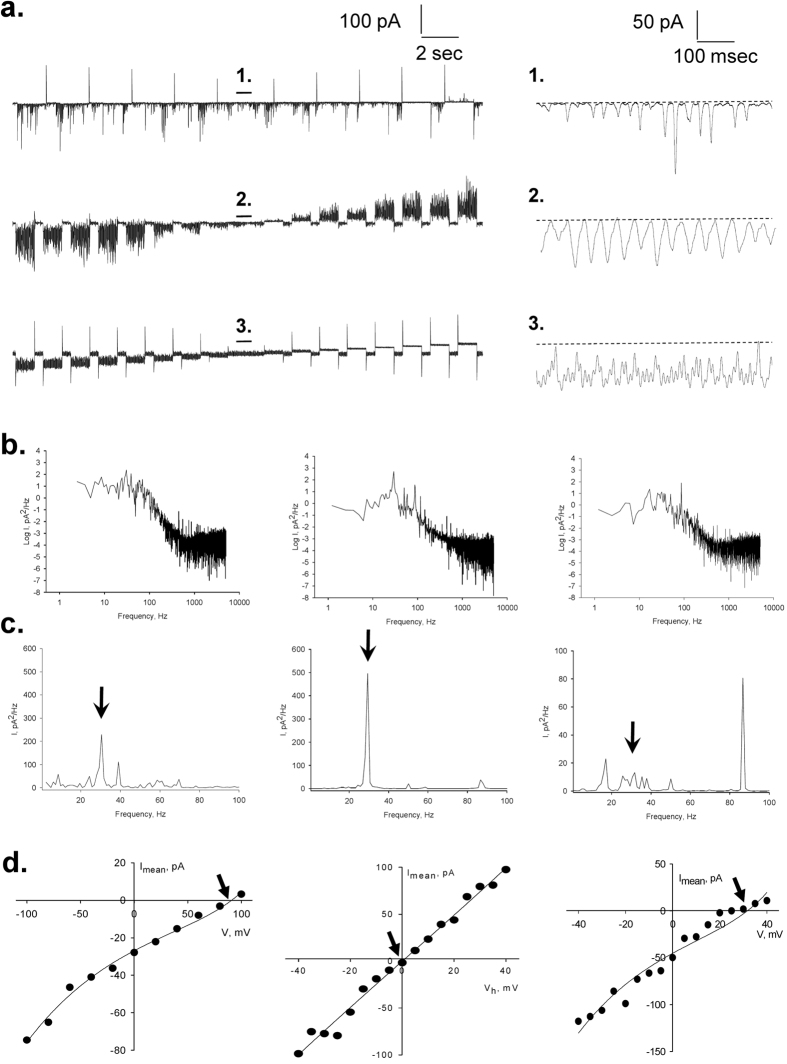
Effect of ionic substitutions on the electrical oscillations of MT sheets. (**a)** *Left*. Representative current tracings obtained as trains of 1.5 sec voltage steps between ±40 mV for biionic asymmetrical conditions, including pipette/bath, in mM: 10 KCl/370 KCl (Top tracing), KCl 140/ NaCl 135 (Middle tracing), and KCl 10/K-gluconate 170 (Bottom tracing). *Right*, Oscillatory currents observed at zero mV for respective conditions. (**b)** Power spectra of oscillatory currents at zero mV, for 10 KCl/370 KCl (*Left*), KCl 140/ NaCl 135 (*Centre*), and KCl 10/K-gluconate 170 (*Right*) conditions, respectively. (**c)** Linear-linear details of Fourier transform are shown for respective conditions. In the presence of K-gluconate in the bath the disappearance of the 29 Hz fundamental frequency and appearance of an 89 Hz peak are noticed. (**d)** Current-to-voltage relationships of mean currents obtained under respective conditions as indicated in (**b**). Reversal potential *V*_*r*_ is indicated with an arrow in each case. Data were successfully fitted with a 2S3B energy model (Black lines).

The ionic selectivity of the oscillatory currents was also explored at different ionic conditions. Increasing the concentration of bathing KCl to 370 mM shifted the reversal potential of the oscillatory currents (Fig. 6a, Top) to 90 mV (Fig. 6d, Left), in agreement with a theoretical Nernst potential for K^+^ (*E*_*K*_ = 92 mV). The cation selectivity of the oscillatory phenomenon was confirmed by increasing K^+^ with a K-gluconate salt (170 mM, Fig. 6a, Bottom). However, under these conditions the reversal potential only shifted to 30 mV (Fig. 6d, Right), suggesting that the counter-lateral anion also affected the oscillatory process. Under these biionic conditions, both amplitude and frequency were also different from those obtained with asymmetrical KCl. This was particularly evident in the frequency domain analysis of the time series, which showed the disappearance of the 29 Hz fundamental frequency and the appearance of a faster, more complex behaviour with a peak at 87 Hz (Fig. 6b, Right). Electrical oscillations in the presence of biionic conditions obtained by isomolar substitution of external KCl by NaCl (Fig. 6a–d, Middle panels) confirmed that the conductive pathway was strictly cation-selective, with a 1:1 K^+^:Na^+^ permeability ratio.

### Voltage oscillations under current clamp conditions

**Figure 7:**
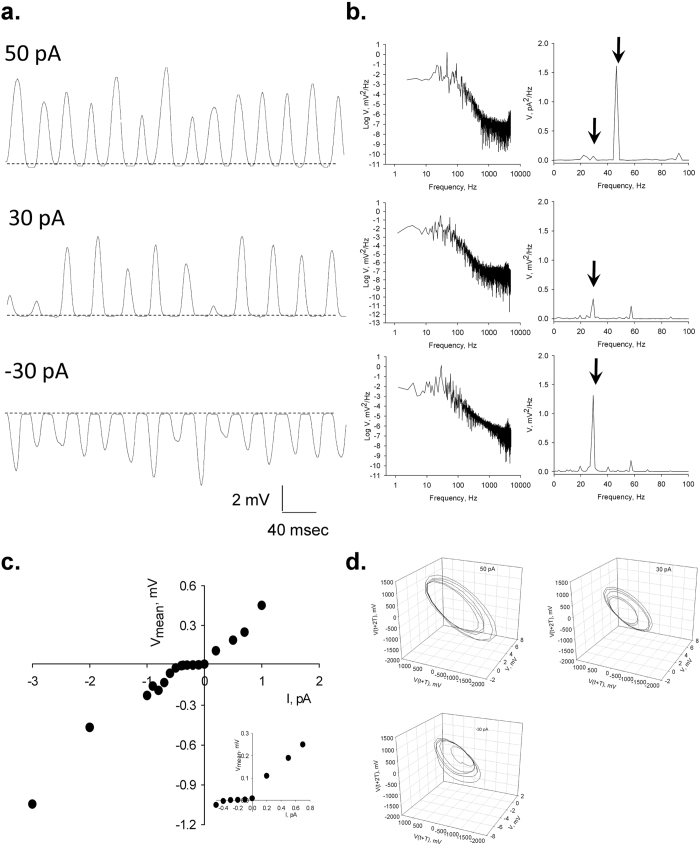
Voltage oscillations under current clamp conditions. (**a)** Voltage oscillations were also observed by current injection under current clamp conditions. Representative tracings are shown for injection of 50 pA (*Top*), 30 pA (*Middle*), and −30 pA (*Bottom*). (**b)** Power spectra showing the voltage oscillations from tracings on Left. Right, Linear-linear detail of Fourier transform that shows the 29 Hz fundamental peak. (**c)** Voltage-to-current relationship for the response at very low current injection stimuli. There is a threshold current at negative but not positive currents, suggesting a gating mechanism disclosed in the negative range (reversal of oscillatory waves). (**d)** Three-dimensional phase-space portraits showing monoperiodic limit cycles. There is a tendency to period doubling at higher holding potentials. Delay time (*T*) for first and second derivatives adopted for phase portraits was 1 ms.

To prove excitability and the possible existence of a gating mechanism for the oscillatory behaviour, we also determined evoked voltage responses under current clamping conditions. Current injection to the patched MT sheet in symmetrical KCl, triggered voltage oscillations (Fig. 7a) with the same fundamental frequency as the current oscillations (Fig. 7b). However, voltage oscillations actually showed sidedness, having a threshold at negative, but not positive currents (Fig. 7c). The current-induced oscillatory voltage behaviour showed clean monoperiodic cycles at different stimuli (Fig. 7d), in agreement with the original current oscillations observed under voltage clamping conditions.

### Effect of taxol on the electrical conductance of MT sheets

**Figure 8:**
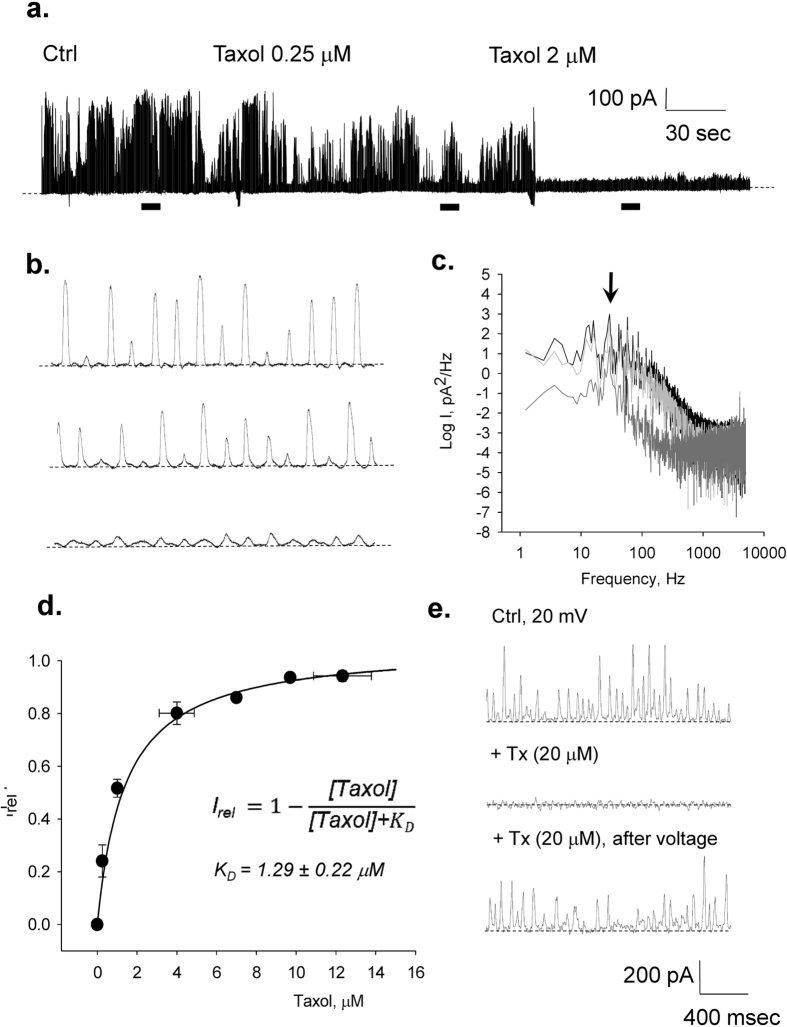
Effect of taxol on the electrical oscillations of MT sheets. The effect of taxol on the electrical current oscillations observed at a holding potential of 10 mV in symmetrical KCl (140 mM). (**a)** Time series of oscillatory currents before and after addition of various concentrations of taxol to the bath, as indicated in the graph. Please note the almost complete disappearance of oscillatory currents after 2 μM taxol. (**b)** Expanded regions (indicated as horizontal bars) of the oscillatory currents shown in (**a**). (**c)** Power spectra of previously expanded regions. Black lines indicate control, Light gray, addition of 0.25 μM taxol, and Dark gray addition of 2.0 μM taxol. (**d)** Dose response curve showing the effect of added taxol on the relative current, calculated as the complement of the remnant relative current. Filled circles represent the mean ± SEM for n = 4. Solid line represents the best Michaelis-Menten fitting to the data with the equation shown in the panel. (**e)** Effect of changes in holding potential on the recovery of electrical oscillations in the presence of taxol.

To explore the possible involvement of the nanopores present in the MT wall in the electrical oscillations, we tested the effect of taxol (Paclitaxel), an anti-mitotic drug, known to diffuse through, and interact with, the nanopores to binding sites in the lumen of intact MTs^38^,^39^. The effect of taxol on the electrical oscillations of the MT sheets was determined under voltage clamping conditions at a holding potential of 10 mV, in symmetrical KCl (140 mM). Addition to the bathing solution of increasing taxol concentrations, elicited a dose-dependent decrease in current amplitude (Fig. 8a,b), which rendered complete inhibition at approximately 10 μM. The changes in amplitude by taxol were not accompanied by any changes in fundamental frequency (Fig. 8c). The change in mean current as a function of bathing taxol (Fig. 8d) followed a pseudo Michaelis-Menten kinetics, with one-binding site and apparent *K*_*D*_ of 1.29 ± 0.22 μM (n = 4). The presence of Zn^2+^ is known to produce anti-parallel 2D sheets^7^,^8^,^9^,^10^, whose symmetry may differ from that of the topological conformation of “microtubule-precursor sheets”^37^. However, the effect of taxol on the commercial tubulin preparation, containing the ion, was identical to the other preparations, suggesting that Zn^2+^ did not affect either the oscillatory behaviour, or the taxol binding properties. Interestingly, the taxol-MT sheet interaction was entirely voltage dependent. An increase in the voltage clamping potential drove the recovery of oscillatory activity with properties similar to those observed under control conditions (Fig. 8e).

## Discussion

The present study provides direct experimental evidence for the electrical properties of the microtubular wall. The surface of the MT behaves as an electrical oscillator that produces ionic currents with variable amplitude and periodicity depending on the driving force and ionic composition. Several aspects of this electrical behaviour could be explored with the high seal patch clamping technique under either voltage or current clamping conditions. The mechanism of oscillation is most consistent with the periodic on-off switching of conductive pores, such that the MT wall behaves as a nonlinear, ion-selective barrier, with transient changes in conductance. The usual response was an organized, self-sustained electrical oscillation that followed the magnitude of the stimulus. Three aspects of the oscillations are relevant, including the amplitude, frequency, and the phase complexity of the periodic cycles.

The initial oscillatory response to voltage in symmetrical KCl most frequently consisted of monoperiodic regimes showing regular sequences of smaller and larger peaks (cycle doubling) at a fundamental frequency of 29 Hz observed in all three preparations tested, namely, both cow and rat brain preparations, and purified commercial tubulin. This fundamental frequency remained remarkably constant at a variety of holding potentials and ionic strength conditions. This was particularly evident during the fast response to short voltage ramp protocols (40 mV/s). Longer, voltage-step protocols in the same range displayed both major changes in amplitude and more complex oscillatory behaviour, but not the fundamental frequency. The similarity among the preparations suggests that MT associated proteins such as MAPs may not play a relevant role in the genesis and/or maintenance of the oscillations, although regulatory role(s) cannot presently be ruled out. Period doubling and more complex behaviours were often spontaneously observed without any deliberate changes in driving force. Three-dimensional phase portraits indicated that period-doubling in the single limit cycles and more complex behaviours were more often observed at negative potentials. Noticeable oscillatory changes such as sudden death disappearance of oscillations and amplitude modulation of the oscillatory responses were prominent in some experiments.

To begin an analysis of the possible mechanistic aspects of the oscillatory phenomenon, several analytical tools applied to ion channels could be of practical use. In fact, the oscillatory electrical currents do behave very much like ion channels that support electrodiffusional cation movements. For example, the mean conductance (charge integration) obtained with voltage step protocols was often linear at all concentrations tested. However, long-standing time series also showed both inwardly and outwardly rectifying properties under symmetrical chemical conditions. Thus, the mean current response to voltage was successfully fitted with a phenomenological model based on Eyring theory^40^, invoking ionic interactions within permeable pores that supported electrodiffusional ion transport. The 2S3B energy model^41^ contemplated two identical energy valleys and three peaks and an interaction factor between ions crossing the conductive pathway. As expected, under symmetrical conditions with a linear response, two identical energy valleys were observed separated by symmetrical electrical distances (Table 2). The model also fitted well several experimental conditions, including rectification patterns under symmetrical ionic conditions, the oscillatory response under various ionic strengths, and the response to different biionic conditions, i.e. K^+^ and Cl^−^ replacements. In particular, the model approximates well the shift in reversal potential after replacement of counter lateral KCl for K gluconate, which cannot be predicted by Nernst equilibrium. Nonlinear conductance patterns, namely rectification under symmetrical conditions, were also well fitted, showing deeply asymmetrical energy profiles, with electrical distances that revealed sites not contemplated in the model (*d*_*x*_ > 1, Table 3). The fitting under all three conditions, symmetrical, outwardly, and inwardly rectifying, were robust and consistent with both cationic selectivity as well as competition of counter lateral ions for putative sites present in the conductive pathway. The model also explained the basic properties of the permeability pathways, and allowed inferring the response to biionic conditions, where counter lateral anions, while not transferred, modify the conductive properties of the pores.

The synchronized electrical currents may be explained by the opening and closing of individual oscillators such as the highly coordinated mechanical changes in tubulin dimers^42^,^43^. The oscillatory behaviour would require a gating mechanism where some sort of electrochemical coupling triggers the opening/closing of the electro-conductive structures. In this context, it is entirely possible that the carboxy termini of the tubulins, which are highly disorganized domains that contribute with a large fraction of the uncompensated charges of the MT, could have a relevant role(s) in the electrical properties of the MTs. However, we have tested the effect of subtilisin on voltage-clamped MT sheets and found no apparent effect at concentrations (10–80 μg/ml) and times (20–120 min) known to cleave the C-termini from the tubulins^44^.

Freedman *et al*.^14^ made a first attempt at modelling the MT wall and ascribing ionic conductances to the nanopores in the MT transmural electrodiffusional transfer of ions. Among the most relevant conclusions of that study were the large cationic conductance of the nanopores, and their role in supporting axial currents along the MT lumen. The data herein support the contention that while the nanopores may be central to the electrodiffusional ion currents, the patches of MT sheets with voltage clamped surface areas of approximately 7–13 μm^2^ displayed large changes in conductance that, in average, would represent an encompassed 3.3 × 10^4^ μm^−2^ synchronized oscillatory units. This parameter would necessarily vary with the size of the patch and, as expected, a correlation was found between patch size (assessed from pipette resistance values before patching), and the current at the onset of patch formation. A limiting function was observed, reaching a constant value as a function of increasing resistance. Thus, once corrected by patch size, current density is a constant parameter largely independent of the size of the surface area, representing the density of functional oscillators in the preparation. Assuming that all nanopores make a contribution to the transmural current, each oscillator would conduct in the range of 0.02–0.14 fA for an applied 5 mV driving force. Consistent with our previous findings^28^,^29^, it could be postulated that localized electrostatic potentials arising from asymmetric ionic distributions between intra- and extra-MT environments, would provide the transmural driving force to support the oscillatory currents. Considering that voltage differences of 1 mV could arise from fluctuations in intracellular K^+^ concentrations of as little as 1.50 mM, oscillatory electrical currents through large open sheets of MTs (as well as cylindrical MTs) may have physiological relevance. Further support for a functional role of the nanopores in the electrical oscillations derives from the inhibitory effect of taxol on the electrical phenomenon, which is in agreement with the fact that the nanopores might be the site(s) of action of antineoplasic drugs such as the taxanes^38^,^39^,^45^. Paclitaxel had an inhibitory effect on the electrical oscillations that followed a pseudo-Michaelis-Menten kinetics with an apparent *K*_*D*_ in the range of 1 μM. This is in agreement with a dissociation constant of 2.5 μM reported for GDP-bound tubulin structures^46^. Interestingly, the inhibitory effect of taxol on the electrical oscillations was entirely reversible upon hyperpolarisation, suggesting the voltage-dependence of this interaction, a particularly appealing modulating factor in the stabilization phenomenon of chemotherapeutic agents on MTs.

The mean conductance from current-to-voltage relationships obtained at the 29 Hz fundamental frequency showed strong nonlinearity, particularly a clear negative resistance region that we modelled with an Esaki diode current equation displaying a “tunnelling” effect that supports oscillatory function^47^. The oscillatory behaviour in our study largely showed a 29 Hz fundamental frequency. Interestingly, several biological oscillatory phenomena prominently display 30 Hz cycles. Mechanical membrane oscillations in this frequency range and involving cytoskeletal structures, have been observed in various cell types^48^,^49^. Another relevant 30 Hz frequency phenomenon is present in the gamma cycle of the brain, the highest frequency brain wave type that ranges between 30–100 Hz^50^. The gamma cycle has been associated with higher cognitive functions, including the formation of ideas, linguistic processing, various types of learning, and meditation^50^,^51^. Gamma waves have been linked to the cognitive act of processing and recall of memories^50^,^51^ and are known to reversibly disappear during anaesthesia-induced deep sleep^52^,^53^, a phenomenon attributed to changes in MT behaviour^54^.

The evidence herein is mechanistically consistent with an organic electrochemical transistor that supports both amplification and self-sustained current (and voltage in this case) oscillations^55^. In this hypothetical mechanism, a gate region of the electrochemical transistor^56^,^57^, most likely due to charge distribution on the MT’s surface^58^ would drive the opening of ion-permeable nanopores that elicit the electrodiffusional circuit. Thus, it is possible that electrostatically-induced vibrations of adjacent αβ tubulin heterodimers may act as electrical oscillators that allow the electrodiffusional ionic transport. This hypothesis will require further investigation.

In conclusion, our findings demonstrate that under physiological conditions, the MT wall behaves as a mesh of highly synchronized electrical oscillators. These MT structures are capable of generating large changes in conductive state. Within the cytoplasm, MT-induced variable currents may contribute to the generation of large intracellular electric fields^59^. Electrical oscillations by MTs may likely play an as yet unknown role in biological signalling events, such as transport of electrical information in neurons, the control of cell division, and the transfer of cargo in MT-driven organelles such as axons, cilia and flagella. Electrically active MTs may be at the centre of a number of MT-associated phenomena ranging from anaesthesia^54^ to the formation of wave patterns in the brain, and the onset of consciousness^60^.

## Methods

### MT isolation from bovine brain

MTs were isolated from bovine brain by cycles of polymerization and depolymerization as described by Ávila *et al*.^61^. Briefly, fresh bovine brains were obtained from a local slaughterhouse, and immediately processed. Brains were rinsed with cold PBS buffer (x3) and maintained at 4 °C throughout the procedure. The brains were chopped and added 1 ml of isotonic buffer per gram of tissue containing: PMSF 1 mM, aprotinin 1 μM, leupeptin 1 μM, and pepstatin 1 μM (MP Biomedicals LLC, Santa Ana, CA). The tissue was homogenized with a blender at low velocity and a Potter homogenizer. The homogenate was centrifuged 30 min at 25,000 *g* (x2). The supernatant was diluted in a solution containing, in mM: MES 100 at pH 6.7, EGTA 2.0, MgCl_2_ 1.0, PMSF 1.0, GTP 1.0, and glycerol 30% (v/v). The mixture was ultra-centrifuged for 45 min at 100,000 *g* at 25 °C. Large two-dimensional MT sheets were easily identified under DIC and immunochemistry (anti-α tubulin antibody, Santa Cruz Biotechnol, Dallas TX) with an Olympus IX71 fluorescence inverted microscope. Samples were kept frozen at −20 °C until further use.

### MT isolation from rat brain

Tubulin from rat brains was obtained as described by Vallee^62^. Briefly, rat brains were harvested from decapitated animals and immediately rinsed and kept in cold distilled water until processing. Brain tissue was homogenized for 4 sec in a blender set at low speed in PEM buffer containing in mM: 100 PIPES (pH 6.95), 2.0 EGTA, and 1.0 MgSO_4_ supplemented with 2-mercaptoethanol and by two passes with a Teflon-in-glass homogenizer. Each aliquot was subjected to 2 passes of the homogenizer at 2000 rpm, and then centrifuged at 23.400 *g* for 90 min in a Sorvall ultracentrifuge with a GSA rotor. The supernatant (cytosolic extract) was decanted. Finally, GTP was added to the extract to a final 1.0 mM concentration and incubated for 24 hours prior to experimentation. Otherwise, samples were kept frozen at −20 °C until further use.

### MT sheets from commercial tubulin

MTs from commercial tubulin were prepared as described by Wolf *et al*.^10^. Briefly, commercial tubulin (TL238-C, Cytoskeleton Inc., Denver CO) was reconstituted at 10 mg/ml as recommended by the manufacturer, and 10 μl of this solution was added to 10 μl of incubation solution containing in mM: MES 20, EGTA 5.0, NaCl 200, MgSO_4_ 1.1, ZnSO_4_ 0.5, and GTP 2.5 (Sigma-Aldrich, St. Louis MO), at pH 5.62. The sample was maintained for 24 hours at room temperature prior to experimentation.

### MT adsorption to APTES-treated glass surfaces

Wherever indicated, MT sheets were deposited onto a glass surface functionalized with a silanization solution containing APTES (3-aminopropyl-triethoxysilane), a silane with an amine group that is positively charged at pH 7^61^. Freshly prepared APTES (0.1%, v/v, 02154766, MP Biomedicals) in distilled water was applied to a clean glass coverslip letting it dry for 10 min before seeding the MT preparation. Approximately 2 μl of either MT sheet preparation was added to the dry surface, letting it rest for 5 min before adding 400 μl of saline solution to fill the patch clamp chamber.

### Electrophysiological data acquisition and analysis

Electrical recordings from voltage-clamped MT sheets were improved such that gigaseal recordings could be obtained (see Table 1). The electronic setup is shown in Fig. 1a, where a conventional patch clamping amplifier (Axopatch 200B, Molecular Devices, Sunnyvale CA), was directly connected to the MT sheet. Briefly, largely 2D-shaped MT sheets were identified in the preparation (Fig. 1b), approached by the patch pipette and sealed by light positive pressure of the tip onto the surface (Fig. 1a,d,e). Seal resistance, and thus the quality of the patch was obtained by imposing 1–5 mV square pulses (Fig. 1e). The pipette tip in solution most often rendered resistances in the order of 5–15 MΩ, as indicated by current deflection and application of Ohm’s law. A decrease in current was a reflection of the increased resistance that reached GΩ values. MT sheet electrical currents where followed by applying different voltage protocols driven from the headstage of the patch-clamp amplifier (Fig. 1a, Right). Usually, the stability of the patch was such that long-lasting experimental conditions could be explored on the MT sheet (Fig. 1f). Patch pipettes were made from soda lime capillary tubes (Biocap, Buenos Aires, Argentina) with 1.25 mm internal diameter. Pipette tips were pulled with a pipette puller (PB-7, Narishige, Tokyo, Japan) and fire polished (MF-9, Narishige, Tokyo, Japan) to a tip diameter of 3–4 μm. Voltage clamp protocols included free-floating at various holding potentials (gap-free protocol), trains of 1500 ms pulses between ±40 mV from a holding potential of zero mV, and 100 and 500 ms ramps within the same voltage range. Very high resistance patches were also subjected to voltage trains between ±100 mV. Current clamping was conducted after zeroing the potential prior to applying the current clamping protocol. Electrical signals were acquired and filtered at 10 kHz, digitized with an analogue-digital converter (Digidata 1440A, Molecular Devices) and stored in a personal computer with the software suite pCLAMP 10.0 (Molecular Devices), also used for data analysis. Sigmaplot Version 10.0 (Jandel Scientific, Corte Madera, CA) was used for statistical analysis and graphics.

### Solutions and chemicals used in electrophysiological experiments

MT sheets were exposed to either symmetrical or asymmetrical saline solutions as indicated: The “intracellular” KCl solution contained, in mM: KCl 140, NaCl 5, EGTA 1.0, and Hepes 10, adjusted to pH 7.18 with KOH. Conversely, the “external” NaCl solution, contained in mM: NaCl 135, KCl 5.0, EGTA 1.0, and Hepes 10, adjusted to pH 7.23 with NaOH. Wherever indicated, anion substitution on either solution was conducted by isomolar replacement with gluconate salt. Biionic conditions were evaluated by aliquots of added KCl stock solution (1 M) to the bathing chamber. Paclitaxel (P3456, Life Technologies, Eugene OR) was prepared as an aqueous solution at a stock concentration of 50 μM.

### Energy modelling of the MT sheet conductance

The nature of the time and voltage-dependent changes of the spontaneous oscillations through the MT wall suggested that the constant field equation of ionic conductance would be unable to model the ionic conductance. Mean data (integrated currents) as a function of the holding potential showed non-ohmic responses. Thus, the mean current-to-voltage (*I*/*V*) relationship of the electrical oscillations obtained from 1.5 s voltage steps responses at various holding potentials under symmetrical ionic conditions, data were fitted with an Eyring multi-barrier rate theory model accounting for the intrinsic rectification and the free energy profile for ion transfer with a three-barrier-two-site (2S3B) minimal conductance model^40^ that supports multiple occupancy and saturation. The model included six energy parameters: three peak energies (*G*_*12*_, *G*_*23*_ and *G*_*34*_), two well energies (*G*_*2*_ and *G*_*3*_), and three electrical distances (*d*_*1*_ to *d*_*3*_), that represent the fraction of the electric field energetically separating peaks and wells. An interaction parameter, *A* = *F*_*out*_/*F*_*in*_, was also included to represent ion-ion interactions, where *F*_*in*_ and *F*_*out*_ are the repulsion factors inside and out the conductive pores, respectively, after ion occupancy. For high activity ranges^41^ the current *I* may be approximated by equation (1):


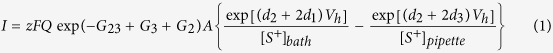


where [S^+^]_*bath*_ and [S^+^]_*pipette*_ are the concentrations of permeable ion in the bath and the pipette, respectively, *V*_*h*_ is the holding potential, *F* is the Faraday constant, and *Q* represents a term enclosing the rate constants between pore states^40^,^41^.

### Tunnel diode current-to-voltage equations

Wherever indicated, the nonlinear mean currents showing negative resistance were fitted to an equation representing the total current *I* of a tunnel diode as given by equation (2):


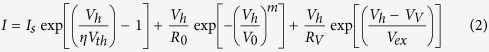


where the terms correspond to the diode, tunnel, and excess currents, respectively. In the context of the MT sheets, all parameters were considered phenomenological constants. *I*_*s*_ is the saturation current, *V*_*h*_ is the holding potential, *η* is the ideality factor and threshold voltage *V*_*th*_ = 10^−3^ V. *R*_*0*_ is the tunnel diode resistance in the ohmic region, and *V*_*0*_ = *V*_*p*_*/*(*1*/*m)*^*1/m*^*, V*_*p*_ is the peak voltage, where *m* = 3. And additional tunnelling current related to parasitic tunnelling via impurities, *I*_*excess*_, is the right-term of the equation, where *V*_*V*_ is the valley voltage, and *R*_*V*_ and *V*_*ex*_ are also empirical parameters.

### Other current and voltage analyses

Unless otherwise stated, electrical tracings shown throughout the study were unfiltered data. Average currents at various holding potentials were obtained by integration of one-second tracings, and expressed as mean ± SEM values, where (n) represented the number of experiments analyzed for a given condition. Power spectra of unfiltered data were obtained by Fourier transform with a subroutine from Clampfit 10.0. Limit cycles were constructed by the time delay (*τ*) approach from the unfiltered tracings, where the lag time *τ* was chosen arbitrarily at 2*f*, where *f* was the sampling frequency of data acquisition. Two-and three dimensional phase space diagrams were constructed in Sigmaplot 10.0.

### Ethical statements

All experimental protocols were approved by the Ethics Committee from the Facultad de Odontología, Universidad de Buenos Aires (approved protocol number 014/14, UBA resolution 0082153/2013). All methods were carried out in accordance with approved guidelines.

## Additional Information

**How to cite this article**: Cantero, M. R. *et al*. Electrical Oscillations in Two-Dimensional Microtubular Structures. *Sci. Rep*. **6**, 27143; doi: 10.1038/srep27143 (2016).

## Figures and Tables

**Table 1:** Mean resistance of tubulin patches.

Sample	Mean resistance ± SEM (MΩ)	Median resistance (MΩ)	N
Commercial tubulin	34.4 ± 3.0	30.4	10
Commercial tubulin (+) APTES	520.8 ± 93.7	396	38
Cow tubulin	49.1 ± 12.0	24.5	55
Cow tubulin (+) APTES	486.8 ± 69.1	400	40
Rat tubulin (+) APTES	755.4 ± 190.9	396	73

**Table 2:** Fitting parameters of Eyring theory (2S3B) model for symmetrical (Ohmic) conductance under symmetrical ionic conditions.

	10 mM KCl	140 mM KCl	500 mM KCl
A	0.23	2.54	45.8
G_2_/G_3_ (*kT*)	−0.49	−0.07	−0.01
G_23_ (*kT*)	0.69	0.23	0.01
d_1_	0.3	0.3	0.3
d_2_	0.4	0.4	0.4
d_3_	0.3	0.3	0.3

**Table 3:** Fitting parameters of Eyring theory (2S3B) model for a symmetrical (non-Ohmic) conductance under symmetrical ionic conditions.

	No rectification	Inward rectification	Outward rectification
A	2.54	0.36	0.42
G_2_/G_3_ (*kT*)	−0.07	−0.77	−0.71
G_23_ (*kT*)	0.23	0.97	−0.71
d_1_	0.3	20.8	3.66
d_2_	0.4	9 × 10^−7^	0.02
d_3_	0.3	7 × 10^−7^	18.7

## References

[b1] Dustin P. Microtubules (Springer Verlag, 1978).

[b2] Kirschner M. & Mitchison T. Beyond self-assembly: from microtubules to morphogenesis. Cell 45, 329–342 (1986). 3516413 10.1016/0092-8674(86)90318-1

[b3] Bray D. Cell Movements. From Molecules to Motility, Part 4, 203–261 (Garland Sci., 1992).

[b4] Desai A. & Mitchison T. J. Microtubule polymerization dynamics. Annu. Rev. Cell Dev. Biol. 13, 83–117 (1997). 9442869 10.1146/annurev.cellbio.13.1.83

[b5] Nogales E., Sharon G. W. & Downing K. H. Structure of the αβ tubulin dimer by electron crystallography. Nature 391, 199–203 (1998). 9428769 10.1038/34465

[b6] Amos L. A. Microtubule structure and its stabilisation. Org. Biomol. Chem. 2, 2153–2160 (2004). 15280946 10.1039/b403634d

[b7] Larsson H., Wallin M. & Edstrom A. Induction of a sheet polymer of tubulin by Zn^2+^. Exp. Cell Res. 100, 104–110 (1976). 945172 10.1016/0014-4827(76)90332-3

[b8] Amos L. A. & Baker T. S. Three-dimensional image of tubulin in zinc-induced sheets, reconstructed from electron micrograph. Intl. J. Biol. Macromol. 1, 146–156 (1979).

[b9] Tamm L. K., Crepeau R. H. & Edelstein S. J. Three-dimensional reconstruction of tubulin in zinc-induced sheets. J. Mol. Biol. 130, 473–492 (1979). 480360 10.1016/0022-2836(79)90435-2

[b10] Wolf S. G., Mosser G. & Downing K. H. Tubulin conformation in zinc-induced sheets and macrotubes. J. Struct. Biol. 111(3), 190–199 (1993). 8003380 10.1006/jsbi.1993.1049

[b11] Chrétien D., Fuller S. D. & Karsenti E. Structure of growing microtubule ends: Two-dimensional sheets close into tubes at variable rates. J. Cell Biol., 129(5), 1311–1328 (1995). 7775577 10.1083/jcb.129.5.1311 PMC2120473

[b12] Nogales E. & Wang H. W. Structural intermediates in microtubule assembly and disassembly: how and why? Curr. Opin. Cell Biol. 18, 179–184 (2006). 16495041 10.1016/j.ceb.2006.02.009

[b13] Wu Z. . Simulations of tubulin sheet polymers as possible structural intermediates in microtubule assembly. PLoS ONE 4(10), e7291 (2009). 19806219 10.1371/journal.pone.0007291 PMC2752796

[b14] Freedman H. . Model of ionic currents through microtubule nanopores and the lumen. Phys. Rev. E Stat. Nonlin. Soft Matter Phys. 81(5 Pt 1), 051912 (2010). 20866266 10.1103/PhysRevE.81.051912

[b15] Pokorný J. Excitation of vibrations in microtubules in living cells. Review. Bioelectrochem. 63 **(1–2)**, 321–326 (2004). 10.1016/j.bioelechem.2003.09.028 15110296

[b16] Kasas S. . Oscillation modes of microtubules. Biol. Cell 96, 697–700, (2004). 15567524 10.1016/j.biolcel.2004.09.002

[b17] Tuszyński J. A., Luchko T., Portet S. & Dixon J. M. Anisotropic elastic properties of microtubules. Eur. Phys. J. E Soft Matter. 17(1), 29–35, (2005). 15864724 10.1140/epje/i2004-10102-5

[b18] Cifra M., Pokornỳ J., Havelka D. & Kučera O. Electric field generated by axial longitudinal vibration modes of microtubule. BioSystems 100, 122–131 (2010). 20178826 10.1016/j.biosystems.2010.02.007

[b19] Sirenko Y. M., Stroscio M. A. & Kim K. W. Elastic vibrations of microtubules in a fluid. Phys. Rev. E 53(1), 1003–1010 (1996). 10.1103/physreve.53.1003 9964335

[b20] Atanasov A. T. Calculation of vibration modes of mechanical waves on microtubules presented like strings and bars. Am. J. Modern Phys. 3(1), 1–11 (2014).

[b21] Tuszynski J. A. . Molecular dynamics simulations of tubulin structures and calculation of electrostatic properties of microtubules. Math. Comp. Model. 41(10), 1055–1070 (2005).

[b22] Stracke R., Böhm K. J., Wollweber L., Tuszynski J. A. & Unger E. Analysis of the migration behaviour of single microtubules in electric fields. Biochem. Biophys. Res. Commun. 293(1), 602–609 (2002). 12054645 10.1016/S0006-291X(02)00251-6

[b23] Minoura I. & Muto E. Dielectric measurement of individual microtubules using the electroorientation method. Biophys. J. 90, 3739–3748 (2006). 16500962 10.1529/biophysj.105.071324 PMC1440755

[b24] Zhao M., Forrester J. V. & McCaig C. D. A small, physiological electric field orients cell division. Proc. Natl. Acad. Sci. USA, 96(9), 4942–4946 (1999). 10220398 10.1073/pnas.96.9.4942 PMC21796

[b25] Zhang P. & Cantiello H. F. Electrical mapping of microtubular structures by surface potential microscopy. Appl. Phys. Lett. 95, 113703 (2009).

[b26] Needleman D. J. . Higher-order assembly of microtubules by counterions: from hexagonal bundles to living necklaces. Proc. Natl. Acad. Sci. USA 101(46), 16099–16103 (2004). 15534220 10.1073/pnas.0406076101 PMC528963

[b27] Zhao Y. & Zhan Q. Electric fields generated by synchronized oscillations of microtubules, centrosomes and chromosomes regulate the dynamics of mitosis and meiosis. Theor. Biol. Med. Model. 9, 26 (2012). 22748065 10.1186/1742-4682-9-26 PMC3503562

[b28] Priel A., Ramos A. J., Tuszynski J. A. & Cantiello H. F. A biopolymer transistor: electrical amplification by microtubules. Biophys. J. 90, 4639–4643 (2006). 16565058 10.1529/biophysj.105.078915 PMC1471843

[b29] Priel A., Ramos A. J., Tuszynski J. A. & Cantiello H. F. Effect of calcium on electrical energy transfer by microtubules. J. Biol. Phys. doi: 10.1007/s10867-008-9106-z (2008). PMC2652550 19669507

[b30] Priel A. & Tuszynski J. A. A nonlinear cable-like model of amplified ionic wave propagation along microtubules. EPL, 83, 68004 (2008).

[b31] Sekulić D. L., Satarić B. M., Tuszynski J. A. & Satarić M. V. Nonlinear ionic pulses along microtubules. Eur. Phys. J. E 34, 49 (2011). 21604102 10.1140/epje/i2011-11049-0

[b32] Henry R., Durai K., Net S., Balraj A. & Priya W. S. Modeling a micro tubule as a diode. J. Biosens. Bioelectron. 2, 106 (2011).

[b33] Sekulić D. L. & Satarić M. V. Microtubule as nanobioelectronic nonlinear circuit. Serbian J. Elect. Engin. 9(1), 107–119 (2012).

[b34] Downing K. H. & Jontes J. Projection map of tubulin in zinc-induced sheets at 4Å resolution. J. Struct. Biol. 109, 152–159 (1992). 1288616 10.1016/1047-8477(92)90046-d

[b35] Li H., DeRosier J., Nicholson W. V., Nogales E. & Downing K. H. Microtubule structure at 8Å resolution. Structure 10, 1317–1328 (2002). 12377118 10.1016/s0969-2126(02)00827-4

[b36] Hamill O. P., Marty A., Neher E., Sakmann B. & Sigworth F. J. Improved patch-clamp techniques for high-resolution current recording from cells and cell-free membrane patches. Pflügers Arch. 391, 85–100 (1981). 6270629 10.1007/BF00656997

[b37] Crepeau R. H., McEwen B. & Edelstein S. J. Differences in α and β polypeptide chains of tubulin resolved by electron microscopy with image reconstruction. Proc. Natl. Acad. Sci. USA 75(10), 5006–5010 (1978). 283410 10.1073/pnas.75.10.5006 PMC336251

[b38] Díaz J. F., Valpuesta J. M., Chacón P., Diakun G. & Andreu J. M. Changes in microtubule protofilament number induced by taxol binding to an easily accessible site. J. Biol. Chem. 273(50), 33803–33810 (1998). 9837970 10.1074/jbc.273.50.33803

[b39] Díaz J. F., Barasoain I. & Andreu J. M. Fast kinetics of taxol binding to microtubules. Effects of solution variables and microtubule-associated proteins. J. Biol. Chem. 278(10), 8407–8419 (2003). 12496245 10.1074/jbc.M211163200

[b40] Cantero M. R. & Cantiello H. F. Effect of lithium on the electrical properties of polycystin-2 (TRPP2). Eur. Biophys. J. 40(9), 1029–1042 (2011). 21678023 10.1007/s00249-011-0715-2

[b41] Hille B. & Schwarz W. Potassium channels as multi-ion single-file pores. J. Gen. Physiol. 72(4), 409–442 (1978). 722275 10.1085/jgp.72.4.409 PMC2228548

[b42] Cifra M., Pokorný J., Jelínek F. & Kučera O. Vibrations of electrically polar structures in biosystems give rise to electromagnetic field: Theories and experiments. PIERS Proc, Moscow, Russia, August 18–21 (2009).

[b43] Kučera O. & Havelka D. Mechano-electrical vibrations of microtubules-Link to subcellular morphology. BioSystems 109, 346–355 (2012). 22575306 10.1016/j.biosystems.2012.04.009

[b44] Sackett D. L. & Wolff J. Proteolysis of tubulin and the substructure of the tubulin dimer. J. Biol. Chem. 261(19), 9070–9076 (1986). 3522582

[b45] Freedman H., Huzil J. T., Luchko T., Ludueña R. F. & Tuszynski J. A. Identification and characterization of an intermediate taxol binding site within microtubule nanopores and a mechanism for tubulin isotype binding selectivity. J. Chem. Inf. Model. 49(2), 424–436 (2009). 19434843 10.1021/ci8003336

[b46] Li Y. K., Edsall R., Jagtap P. G., Kingston D. G. I. & Bane S. Equilibrium studies of a fluorescent paclitaxel derivative binding to microtubules. Biochem. 39, 616–623 (2000). 10642187 10.1021/bi992044u

[b47] Seabaugh A. & Lake R. Tunnel diodes. Encyclop. App. Phys. 22, 335–359 (1999).

[b48] Korenstein R. & Levin A. Membrane fluctuations in erythrocytes are linked to MgATP-dependent dynamic assembly of the membrane skeleton. Biophys. J. 60, 733–737 (1990). 1932557 10.1016/S0006-3495(91)82104-X PMC1260118

[b49] Tuvia S., Bitler A. & Korenstein R. Mechanical fluctuations of the membrane-skeleton are dependent on F-actin ATPase in human erythrocytes. J. Cell Biol. 141, 1551–1561 (1998). 9647648 10.1083/jcb.141.7.1551 PMC2133013

[b50] Jensen O., Kaiser J. & Lachaux J. P. Human gamma-frequency oscillations associated with attention and memory. Review. Trends Neurosci. 30(7), 317–324 (2007). 17499860 10.1016/j.tins.2007.05.001

[b51] Miltner W. H. R., Braun C., Arnold M., Witte H. & Taub E. Coherence of gamma-band EEG activity as a basis for associative learning. Nature, 397, 434–436 (1999). 9989409 10.1038/17126

[b52] Vanderwolf C. H. Are neocortical gamma waves related to consciousness? Brain Res. 855(2), 217–224 (2000). 10677593 10.1016/s0006-8993(99)02351-3

[b53] Ma J., Shen B., Stewart L. S., Herrick I. A. & Leung L. S. The septohippocampal system participates in general anesthesia. J. Neurosci. 22, RC200 1–6 (2002). 11784812 10.1523/JNEUROSCI.22-02-j0004.2002 PMC6758657

[b54] Emerson D. J. . Direct modulation of microtubule stability contributes to anthracene general anesthesia. J. Am. Chem. Soc. 135(14), 5389–5398 (2013). 23484901 10.1021/ja311171u PMC3671381

[b55] Tu D. & Forchheimer R. Self-oscillation in electrochemical transistors: An RLC modeling approach. Solid-State Electronics, 69, 7–10 (2012).

[b56] Shinwari M. W., Deen M. J. & Landheer D. Study of the electrolyte-insulator-semiconductor field-effect transistor (EISFET) with applications in biosensor design. Microelectron. Reliab. 47(12), 2025–2057 (2007).

[b57] Bernards D. A. & Malliaras G. G. Steady-state and transient behavior of organic electrochemical transistors. Adv. Funct. Mater. 17, 3538–3544 (2007).

[b58] Baker N. A., Sept D., Simpson J., Holst M. J. & McCammon J. A. Electrostatics of nanosystems: Application to microtubules and the ribosome. Proc. Natl. Acad. Sci. USA, 98(19), 10037–10041 (2001). 11517324 10.1073/pnas.181342398 PMC56910

[b59] Tyner K. M., Kopelman R. & Philbert M. A. “Nanosized voltmeter” enables cellular-wide electric field mapping. Biophys. J. 93, 1163–1174 (2007). 17513359 10.1529/biophysj.106.092452 PMC1929021

[b60] Hameroff S. & Penrose R. Orchestrated reduction of quantum coherence in brain microtubules: A model for consciousness. Math. Comp. Simul. 40, 453–480 (1996).

[b61] Ávila J., Soares H., Fanarraga M. L. & Zabala J. C. Isolation of microtubules and microtubule proteins. Curr. Protoc. Cell Biol. 39, 3.29.l–3.29.28 (2008). 10.1002/0471143030.cb0329s39 18551420

[b62] Vallee R. B. Reversible assembly purification of microtubules without assembly-promoting agents and further purification of tubulin, microtubule-associated proteins, and MAP fragments. Meth. Enzymol. 134, 89–104 (1986). 3821583 10.1016/0076-6879(86)34078-3

